# Retrograde tracing and toe spreading after experimental autologous nerve transplantation and crush injury of the sciatic nerve: a descriptive methodological study

**DOI:** 10.1186/1749-7221-7-5

**Published:** 2012-04-30

**Authors:** Sabien GA van Neerven, Ahmet Bozkurt, Dan Mon O’Dey, Juliane Scheffel, Arne H Boecker, Jan-Philipp Stromps, Sebastian Dunda, Gary A Brook, Norbert Pallua

**Affiliations:** 1 Department of Plastic Surgery, Reconstructive and Hand Surgery, Burn Center, Medical Faculty, RWTH Aachen University, Pauwelsstrasse 30, 52074 Aachen, Germany; 2 Institute of Neuropathology, Medical Faculty, RWTH Aachen University, Aachen, Germany

**Keywords:** Peripheral nerve injury, Repair strategy, Peripheral nerve regeneration, Neurotmesis, SSI, Sciatic nerve injury, Rat model

## Abstract

Evaluation of functional and structural recovery after peripheral nerve injury is crucial to determine the therapeutic effect of a nerve repair strategy. In the present study, we examined the relationship between the structural evaluation of regeneration by means of retrograde tracing and the functional analysis of toe spreading. Two standardized rat sciatic nerve injury models were used to address this relationship. As such, animals received either a 2 cm sciatic nerve defect (neurotmesis) followed by autologous nerve transplantation (ANT animals) or a crush injury with spontaneous recovery (axonotmesis; CI animals). Functional recovery of toe spreading was observed over an observation period of 84 days. In contrast to CI animals, ANT animals did not reach pre-surgical levels of toe spreading. After the observation period, the lipophilic dye *DiI* was applied to label sensory and motor neurons in dorsal root ganglia (DRG; sensory neurons) and spinal cord (motor neurons), respectively. No statistical difference in motor or sensory neuron counts could be detected between ANT and CI animals.

In the present study we could indicate that there was no direct relationship between functional recovery (toe spreading) measured by SSI and the number of labelled (motor and sensory) neurons evaluated by retrograde tracing. The present findings demonstrate that a multimodal approach with a variety of independent evaluation tools is essential to understand and estimate the therapeutic benefit of a nerve repair strategy.

## **Introduction**

Following peripheral nerve injury (PNI), autologous nerve transplantation (ANT) is still favoured as the gold standard to bridge nerve gaps, when tensionless end-to-end nerve coaptation is not possible [1]. This type of nerve lesion is the foremost challenging form of PNI to repair, since complete nerve transection (neurotmesis) does not only include a disruption of axons, but also of the nerve trunk itself including its connective tissue elements (i.e. epi-, peri- and endoneurium). In such cases, directed axonal regeneration with functional recovery of the end-organs is not possible without a nerve repair strategy, which includes reconstruction of these elements [2,3]. So far, ANT provides the best cellular, molecular, and structural composition to support axonal regeneration. However, ANT is associated with comorbidities at the harvest site of the donor nerve, while the amount of available donor nerves is limited [4,5].

Over the recent decades, a large number of approaches have been made to develop bioartificial nerve guides as an alternative to ANT. As such, non-nervous autologous materials, allogenic, xenogenic or synthetic materials have been tested *in vitro* and *in vivo *[5]. Complete transection (neurotmesis-model) of the rat sciatic nerve is frequently used as an animal model to study the efficacy of nerve repair strategies, and in such experiments ANT is often used as a positive control (growth supporting substrate) for the other experimental groups (i.e. bioartificial nerve guides). Furthermore, the therapeutic efficacy of such bioartificial nerve guides has been evaluated by a number of methods. These include methods to study regenerative processes, mostly by measuring structural/histomorphometric parameters and/or methods to study functional properties using electrophysiological measures or behavioural tests [6,7]. Frequently used behavioural tests include the static sciatic index (SSI) [8,9], sciatic functional index (SFI) [10,11], stance factor [12], ankle kinematics [13] toe out angle (TOA) [14], and the Catwalk gait analysis [15]. In addition, the degree of axonal regeneration can be provided by morphometric parameters such as nerve fiber density, axonal diameter, myelin sheath thickness and g-ratio, as well as retrograde tracing experiments [4,16,17]. In particular, retrograde tracing experiments indicate the type of axons (from motor [spinal cord] *versus* sensory neurons [dorsal root ganglia]) that regenerate. Yet, none of the individual methods cover all the possible therapeutic effects of peripheral nerve injury repair. As such, combinations of the different, but complementary methods have been applied [15].

The goal of the present study is to analyse the relationship between frequently used tracing methods that estimate the degree of structural regeneration and behavioural tests that evaluate the extent of functional recovery in peripheral nerve regeneration. We therefore used different techniques to examine and determine the degree of regeneration of the rat sciatic nerve after a standard procedure, i.e. transection followed by ANT repair. It cannot be highlighted enough that axonal regeneration is required for functional recovery, though without appropriate functional benefit, any structural regeneration is meaningless. Consequently, to evaluate this functional-structural relationship after ANT repair, functional recovery was determined by visual SSI, whereas structural regeneration was analysed by means of retrograde tracing experiments. In addition, a reproducible and standardized sciatic nerve crush injury (axonotmesis model) [15] was used as a positive control experiment, since successful axonal regeneration and functional repair occurs spontaneously after this type of injury [18].

## **Material and methods**

### **Animals**

All animals were maintained in accordance with the guidelines of the German animal protection statute and experimental protocols were approved by the governmental review committee.

In this study, every attempt was made to minimize the number of animals as well as any pain and discomfort. In total of 25 female Lewis rats (Charles River, Germany) of approximately 220 g were housed under temperature controlled conditions of 21 ± 1°C, with normal 12:12 h light/dark cycle and *ad libitum* access to food and water.

### **Surgical procedure**

Prior to surgery, animals received a subcutaneous injection of 150 μg/kg bw Buprenorphinhydrochlorid (Temgesic®). Subsequently, anaesthesia was induced by 5% isoflurane/air mixture and maintained on 2.5% isoflurane/air. The right thigh and leg were shaved, disinfected and animals were then placed in abdominal position on a sterile operation field. A skin incision of approximately 3 cm was made over the gluteal region exposing the right sciatic nerve from the sciatic notch to the point of trifurcation. The ischiocrural musculature was prepared with minimal tissue damage to ensure optimal conditions for complete functional recovery.

For ANT, the sciatic nerve of 15 animals was completely excised over a distance of 2 cm. This nerve segment was removed and immediately re-implanted and sutured with three 10–0 epineurial single stitches (Ethilon, Ethilon Inc., Sommerville USA). As a positive control experiment, 10 animals received a sciatic nerve crush injury (axonotmesis). The crush injury was made using a sterile non-serrated clamp (kindly provided by Prof. Geuna, University of Torino, Italy: original source: Institute of Industrial Electronic and Material Science, University of Technology, Vienna, Austria) [18,19]. This clamp yielded a constant force of 54 N (pressure of p = 9 MPa) over a time period of 30 s. The clamp was positioned approximately 10 mm above the point of trifurcation of the sciatic nerve. The skin was subsequently closed with 4–0 single suture stitches (4–0 Prolene®, Ethicon Inc., Somerville). The left sciatic nerve was left intact and served as an internal (intra-individual) control.

### **Toe spreading**

To determine pre-operative toe spreading, animals were placed into acrylic glass containers (20 cm × 12 cm × 9 cm) on a transparent base plate, as previously described [9,15]. A webcam (Logitec QuickCamPro4000) was positioned underneath the transparent base plate in order to photograph the plantar surface of the animal’s paws. This webcam was connected to a computer running the custom-made image acquisition program (“Visual-SSI”; [9]). Subsequently images were analysed with the freely available image editing software AxioVision LE Rel. 4.4 (Zeiss, Jena Germany) for the quantification of toe spreading. Toe-spread-factors were determined by measuring the distance between the first and fifth toe (1–5, toe spread) and between the second and fourth (2–4, intermediate toe spread) as previously described by *Bervar (2000*) [8].

(1) $$\begin{array}{l} \text{TSF}=\text{OTS}-\text{NTS}/\text{NTS}\\ \text{ITSF}=(\text{OITS}-\text{NITS})/\text{NITS} \end{array}$$

Abbreviations: TSF = toe spread factor; OTS = operated side toe spread; NTS = non-operated side toe spread; ITSF = intermediate toe spread factor; OITS = operated side intermediate toe spread; NITS = non-operated side intermediate toe spread

Measurements were made by a single blinded observer. After surgery, toe spreading was analyzed once a week up to 84 days post-operation (dpo).

### **Tracing experiments**

The lipophilic dialkycarbo-cyanine DiI (1,1′-dioctadecyl- 3,3,3′,3′-tetramethylindocarbocyanine perchlorate, Molecular Probes, Leiden, The Netherlands) was used for retrograde tracing of DRG sensory and spinal cord motor neurons [16,20,21]. Twelve weeks after surgery, the sciatic nerves were re-exposed and the epineurium was opened. DiI crystals were applied (+/− 2 mm) distal to the distal coaptation or crush site (marked by ethilon 9.0 non-absorbable sutures). After two further weeks, animals were transcardially perfused with 4% PFA followed by explantation and post-fixation of L3-L6 of the spinal cord and associated DRGs [22]. Cryoprotection was performed by transferring the tissue into 10% sucrose, followed by 30% sucrose (both at 4°C) and freezing at −80°C. Preparations were subsequently embedded in Tissue-Tek® and cryosectioned in slices of 25 μm (DRG) and 50 μm (spinal cord) thickness, which were mounted on Superfrost Cold Plus Gold glass slides and stored at 4°C for 30 minutes. Sections were then observed under a fluorescent microscope. Every sixth slide of the DRG and every third slide of the spinal cord was used for counting labelled profiles to prevent double counts. Fluorescent perikarya were assessed with the freely available image-editing program AxioVision LE (Rel. 4.4, Zeiss, Jena Germany). Neuronal profiles were counted positive only when a clear fluorescent signal was detectable (characterized by a bright fluorescent red cytosol, with clear nucleus). To minimize observer-dependent errors all sections were analysed by one single observer.

### **Statistical analysis**

All graphs represent means with standard error of the mean (SEM). P-values of less than 0.05 were considered as statistically significant and significance levels were marked with * when *p*<0.05 or ** when *p*<0.01. Differences in retrograde tracing were examined by the paired Student’s t- test. Furthermore, post-operative changes of TSF and ITSF compared to pre-operative values were analysed by one-way analysis of variance with repeated measures, followed by Dunnett’s post hoc multiple comparisons test. Data are expressed as change from baseline up to 84 days after injury. At least 10 images per animal were analyzed at every time point.

## **Results**

Animals quickly recovered from surgery and showed no drop in bodyweight, reflecting their good general condition afterwards. Animals did not show any signs of autotomy behaviour throughout the study.

**Figure 1:**
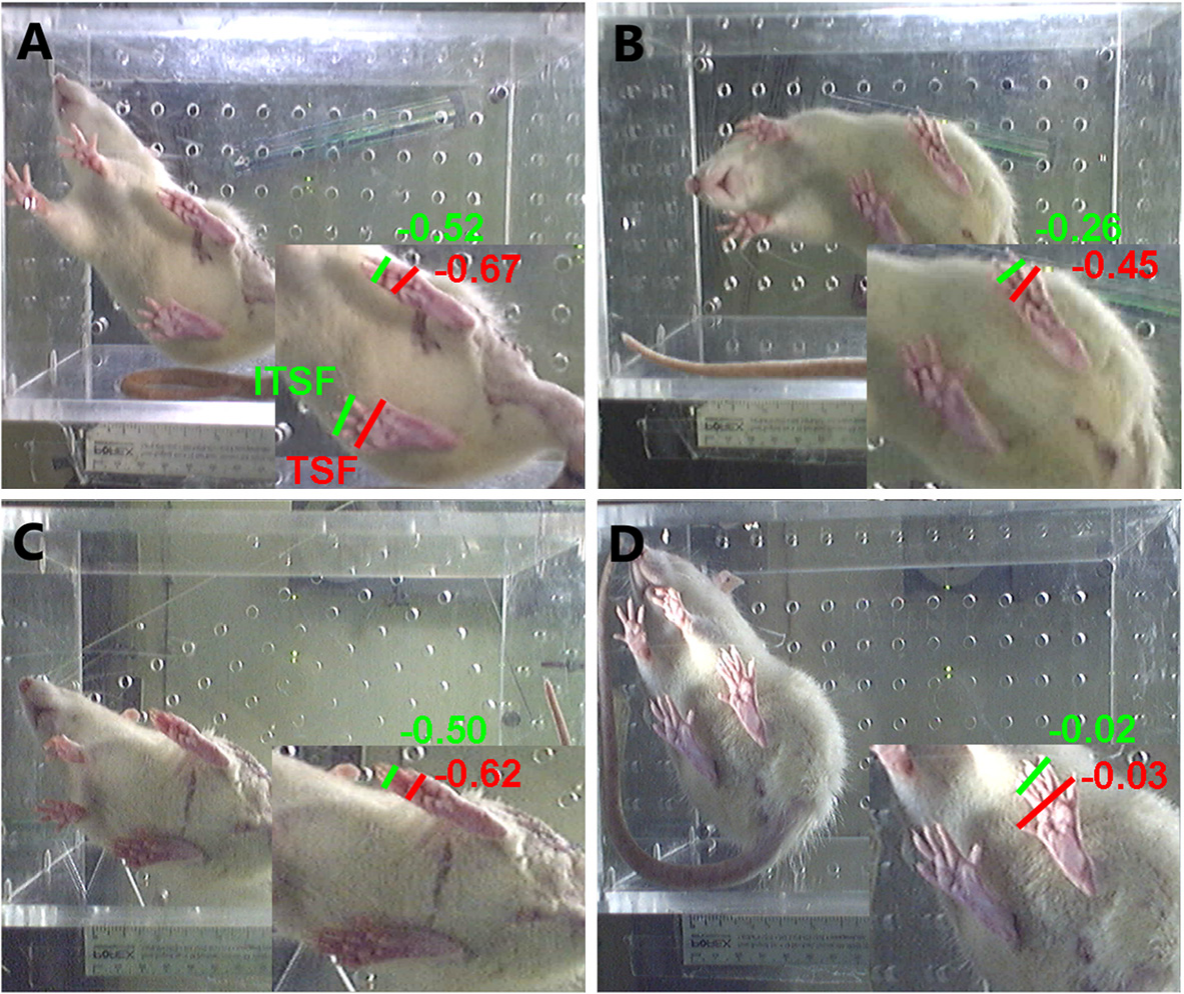
**Functional regeneration after rat sciatic nerve injury.** Original pictures, demonstrating the plantar surface view of the rat’s hind paws. Left hind paw represents the healthy (contralateral) paw, and right hind paw (ipsilateral) the operated side (The camera inverted the view on the animal, **A-D**). Accordingly paw print parameters were analyzed by measuring toe spread factor (TSF) digits 1–5, and intermediate toe spread factor (ITSF) digits 2–4. Typically the posture of the ipsilateral paw is devoid of any toe spreading during the first week after sciatic nerve injury (**A, C**). At 84 dpo toe spreading was significantly improved in both groups (**B, D**), but the ANT group did not reach pre-surgical values.

*Functional regeneration* was analysed by measuring toe spreading and calculation of toe spread factor (TSF) and intermediate toe spread factor (ITSF) of the left (contralateral to the lesion site) and right (ipsilateral to the lesion site) paws of animals after ANT or CI (Table 1). One week after surgery, both groups (ANT and CI animals) showed loss of toe spreading (Figure 1A, C). During the observation period, toe spreading improved significantly over time (Figure 1B, D and Table 1). Although improvement was substantial, the ANT group did not reach pre-surgical levels of toe spreading at the end of the observation period (Table 1, pre-operative: TSF:−0.03 ± 0.03 ITSF:−0.06 ±0.05 and 84 dpo: TSF:−0.45 ±0.04, ITSF:−0.26 ± 0.05). In contrast, positive control CI animals recovered completely from injury (Table 1, pre-operative: TSF:−0.01 ± 0.04 ITSF:−0.03 ± 0.05 and 84 dpo: TSF: 0.03 ± 0.04, ITSF:−0.02 ± 0.05, *p*<0.01).

**Table 1:** Toe spreading after sciatic nerve injury

**Time (dpo)**	**ANT (TSF)**	**CI (TSF)**	**ANT (ITSF)**	**CI (ITSF)**
pre-operative	−0.03 ± 0.03	−0.01 ± 0.04	−0.06 ±0.05	−0.03 ± 0.05
7	−0.67 ±0.02	−0.62 ± 0.02	−0.52 ±0.03	−0.50 ± 0.03
35	−0.49 ±0.02	−0.16 ± 0.04 **	−0.39 ± 0.04	−0.12 ± 0.05 **
84	−0.45 ±0.04	0.03 ± 0.04 **	−0.26 ± 0.05	−0.02 ± 0.05 **

Toe spreading was measured pre-operative and at different time points after autologous nerve transplantation (ANT) or crush injury (CI). CI and ANT treated animals both demonstrated improvement of toe spread factor (TSF) and intermediate toe spread factor (ITSF) over time. Although both groups reached a plateau at approximately 35 dpo, ANT treated animals never reached pre-operative values, whereas CI animals did (** *p*<0.01).

**Figure 2:**
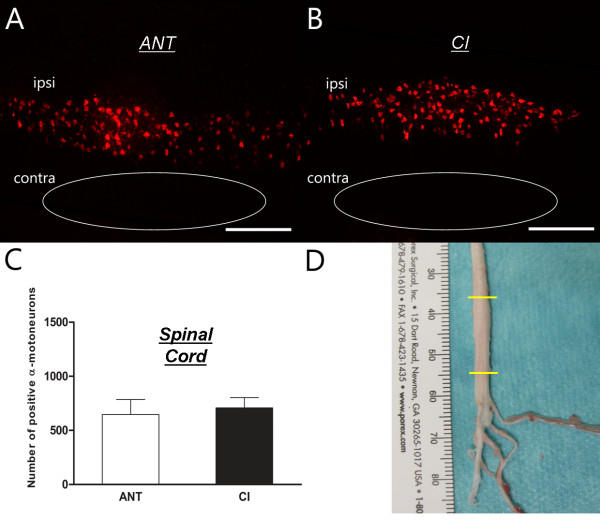
**Retrograde tracing - α-motor neurons:** Images of ANT (**A**) and CI (**B**) lumbar spinal cord containing retrograde traced α-motor neurons. Quantification of positively traced neurons revealed no difference in the amount of labelled α-motoneurons between ANT and CI animals (**C**). Total amount of positively traced motor neurons was counted in lumbar spinal cord segments L3-L6 (**D**).

**Figure 3:**
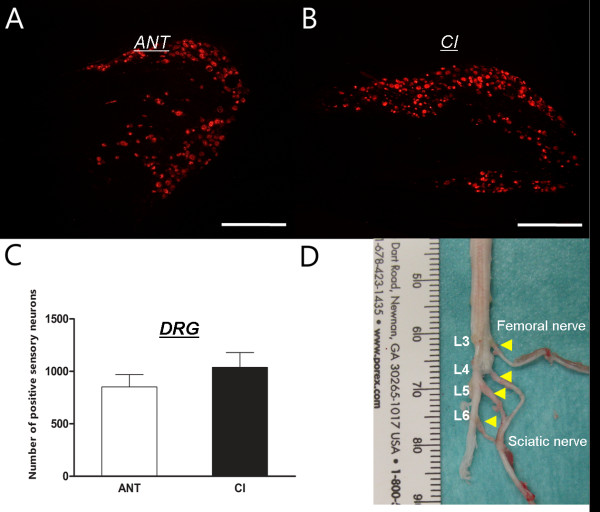
**Retrograde tracing - sensory neurons:** Images of ANT (**A**) and CI (**B**) lumbar DRGs containing retrograde traced sensory neurons. Quantification of positively traced neurons revealed no difference in the amount of labelled sensory neurons between ANT and CI animals (**C**). Total amount of positively traced motor neurons was counted in DRGs L3-L6 of the lumbar spinal cord (**D**).

Retrograde tracing with DiI was used to evaluate *structural* aspects of sciatic nerve regeneration. Representative images and quantification of positively labelled neurons in the spinal cord and DRGs are shown in Figures 2 and 3. Longitudinal sections of the ventral columns revealed positively labelled perikarya in the spinal cord ipsilateral to the lesion site. Logically, α-motor neurons of the contralateral side (below) were not stained (Figure 2A, B). ANT animals showed a trend towards lower numbers of positively stained motoneurons compared to CI animals (646 ± 139 versus 708 ± 95), but this difference was not statistically significant (Figure 2C, p>0.05). DRGs of spinal levels L3-6 showed a similar tendency between ANT and CI animals (Figure 3A, B). ANT animals (851 ± 118) showed less positively traced sensory neurons compared to CI (1038 ± 141), but again, this difference was not statistically significant (Figure 3C, p>0.05). Additionally, preferential regeneration of sensory or motor fibers in ANT or CI animals was neither observed (p>0.05).

## **Discussion**

This present study indicates a discrepancy between functional recovery evaluated by toe spreading and structural regeneration analyzed by retrograde tracing. Toe spreading of ANT animals improved significantly over time, but did not reach pre-surgical values after 12 weeks of recovery. In contrast, CI animals recovered quickly after PNI with a degree of toe spreading comparable to pre-surgical values (Table 1, Figure 1). Interestingly, this difference in functional recovery evaluated by visual SSI could not be corroborated by a structural difference, since the amount of traced α-motor or DRG sensory neurons were equal in both CI and ANT groups.

Ideally, peripheral nerve regeneration and the therapeutic effect of a repair strategy should be estimated in an experimental setup where functional recovery and structural regeneration can be studied simultaneously. Electrophysiological measurements are frequently used to evaluate the degree of reinnervation by determining signal transduction properties of newly regenerated nerve fibers [23-25]. These parameters, however, give mainly an impression purely based on the physical condition of the regenerated nerves and do not predict the benefit on motor and sensory function [25]. In contrast, behavioural analyses add lacking information on the functional effect of a repair strategy. Among the large variety of available behavioural tests, SSI has been proven to be a reliable, valid and highly efficient method to determine functional recovery after PNI [8,9,15].

In addition to behavioural tests, histomorphometric analyses and nerve tract tracing experiments are frequently used to analyse axonal regeneration [9,15,17]. Parameters such as axon density and axon diameter give information about quantity of regeneration, whereas parameters like myelin sheath thickness and g-ratio (inner axon diameter/overall axon diameter) provide details about the quality and maturity of the regenerated axons. Yet, axon densities may be overestimated when local aberrant sprouting occurs [26], and axons *in situ* are counted as multiple positive units while they are actually derived from one single sensory or motor branch. Moreover, morphometric measurements do not provide any information about the type (sensory or motor) of axon that has been analyzed. Retrograde tracing experiments are, therefore, frequently used to supplement morphometric analyses, because they provide additional information about the origin of the regenerated axons. Lipophilic dyes such as DiI are applied at the distal part of the sciatic nerve from which the substance is retrogradely transported along the sensory or motor branches to their origin (i.e. neurons located in DRG and spinal cord) [16,20,21]. The number of labelled neurons can be determined to provide information about the amount of regenerated axons without being subjected to sprouting events at the lesion site.

In the present methodological study, we investigated whether a relationship exists between the number of positively labelled sensory and motor neurons and the degree of functional recovery (toe spreading measurements) after sciatic nerve injury in the rat. Our results demonstrate that ANT animals do not show the same extent of functional recovery as CI animals. Toe spreading (Table 1) and previous published SSI values improved in time in both groups and reached a plateau around 35 dpo that was maintained until the end of the observation period (84 dpo). Yet, CI animals reached pre-surgical toe spread values whereas ANT animals did not [27]. One could expect that this functional discrepancy coincides with a difference in structural regeneration [27]. Interestingly, the difference in toe spreading between the two groups could not be addressed to a deviation in the amount of retrograde traced neurons. This discrepancy between functional recovery and the amount of positively labelled neurons points out that interpretations and conclusions on structural data generated by retrograde tracing experiments in relation to functional measurements should be made with caution. Others studies demonstrate a comparable structural-functional inconsistency [28,29]. Not identical, but a similar difference between the degree of functional recovery and the number of retrograde traced neurons has previously been published in a study of Tomov et al., 2002 where blind and visually normal rats underwent transection of the facial nerve. Both groups showed recovery of whisker motion in time, yet normal visual rats developed spasticity, whereas blind rats completely recovered from injury. Recovery of whisker motion was shown to be independent of the amount of positively traced motor neurons in the facial nucleus in both groups. The authors concluded that behavioural demand and forced overuse could explain the differences in motor performance observed [30].

The functional difference between our experimental groups cannot be explained by selective regeneration of sensory or motor axons induced by ANT repair, since retrograde labelling demonstrated regeneration of both fiber types. Yet, the value of DiI tracing experiments is restricted to these two fiber classifications, since they do not provide any information about what subtype of sensory (i.e. Aβ, C-fibers or Aδ) or motor (α/γ motor neurons) fibers regenerate.

First requirement for efficient regeneration and functional improvement is the intrinsic ability of axons to spontaneously start re-growing after PNI. Secondly, directed and sustainable axonal growth across the nerve gap is necessary. Eventually the axons that cross the nerve gap should be able to make functional contacts that are myotopically organized to properly re-innervate their target organs. Functional regeneration often fails if these requirements are not achieved and if processes such as axonal misdirection, aberrant sprouting of nerve fibers or polyneural innervation occurs [26]. Interestingly, previously published morphometric analyses of our group revealed that nerves derived from ANT animals showed remarkable morphological differences characterized by increased amounts of axons compared to CI. In addition, axon diameter and myelinated axon diameter were both smaller in the ANT group compared to the CI group. Substantial differences in myelin related parameters could also be observed. This was mainly reflected by the g-ratio (inner axon diameter/ myelinated axon diameter), which was less in ANT animals [27]. This larger number of small diameter axons which were of a more immature phenotype, indeed suggest that aberrant sprouting occurs after ANT repair. Our tracing experiments do not provide any information about the latter, nor do they provide any information about nerve-muscle interaction. Electrophysiological experiments, where muscle potentials and nerve conductance are evaluated, provide this lacking information. As such, peripheral nerve repair can only be evaluated properly by a multimodal approach with complementary techniques examining the relationship between functional recovery and structural regeneration.

## **Conclusion**

Our present data indicate that the level of functional recovery observed is not directly correlated to the amount of retrograde labelled sensory or motor neurons in DRGs and spinal cord. As such, our data may indicate that an explanation for the functional difference between the groups could be found in processes that occur beyond the distal coaptation site. As such, multimodal approach is essential in the evaluation of peripheral nerve regeneration. The different approaches used in this present study shed light on specific aspects of peripheral nerve regeneration, but obviously also show their limitations in this respect. So when the impact of alternative nerve guides is tested, elucidation of structural-functional correlation is mandatory.

## Competing interests

We declare that the interpretation of our data or the presentation of the comprehending information, are not influenced by a personal or financial relationship with other people or organizations. In the past five years we did not receive any reimbursements, fees, funding or salary from an organization that may in any way gain or lose financially from the publication of this manuscript, either now or in the future. We declare that we do not hold any stocks or shares in an organization that may in any way gain or lose financially from the publication of this manuscript, either now or in the future. We declare that we are not currently applying for any patents relating to the content of the manuscript. We declare that we do not have any other financial competing interests. Additionally, we declare that there are no non-financially competing interests (i.e. political, personal, religious, ideological, academic, intellectual, commercial or any other).

## Authors’ contributions

SvN: has made contributions to the statistical analysis and interpretation of the data and has been involved in drafting and critically revising the manuscript. AB: has made contributions to the concept and design of the study, performed the animal operations and has been involved in drafting and critically revising the manuscript. DMO’D: has made contributions to the analysis and interpretation of the data. JS: has been involved in the acquisition of the data by performing the behavioral experiments (SSI). AHB: has been involved in the acquisition of the data by performing the behavioral analysis and the evaluation of the tracing experiments. JPS: has made contributions to the analysis and interpretation of the data. SD: has made contributions to the analysis and interpretation of the data. GB: has been involved in drafting and critically revising the manuscript. NP: has made contributions to the concept and design of the study and has been involved in drafting and critically revising the manuscript. All authors read and approved the final manuscript.
